# Recent State of Wearable IMU Sensors Use in People Living with Spasticity: A Systematic Review

**DOI:** 10.3390/s22051791

**Published:** 2022-02-24

**Authors:** Yehuda Weizman, Oren Tirosh, Franz Konstantin Fuss, Adin Ming Tan, Erich Rutz

**Affiliations:** 1 Department of Health and Medical Sciences, School of Health Sciences, Hawthorn Campus, Swinburne University of Technology, Melbourne 3122, Australia; otirosh@swin.edu.au (O.T.); amtan@swin.edu.au (A.M.T.); 2 Chair of Biomechanics, Faculty of Engineering Science, University of Bayreuth, D-95440 Bayreuth, Germany; franzkonstantin.fuss@uni-bayreuth.de; 3 Department of Orthopaedics, The Royal Children’s Hospital, Melbourne 3052, Australia; erich_rutz@hotmail.com; 4 Murdoch Children’s Research Institute, MCRI, Parkville, Melbourne 3052, Australia; 5 Department of Paediatrics, The University of Melbourne, Melbourne 3052, Australia; 6 Medical Faculty, University of Basel, 4001 Basel, Switzerland

**Keywords:** spasticity, spasticity assessment, wearable devices, inertial measurement unit (IMU) sensors, neurological disorder

## Abstract

Spasticity is a disabling characteristic of neurological disorders, described by a velocity-dependent increase in muscle tone during passive stretch. During the last few years, many studies have been carried out to assess spasticity using wearable IMU (inertial measurements unit) sensors. This review aims to provide an updated framework of the current research on IMUs wearable sensors in people living with spasticity in recent studies published between 2017 and 2021. A total of 322 articles were screened, then finally 10 articles were selected. Results show the lack of homogenization of study procedures and missing apparatus information in some studies. Still, most studies performed adequately on measures of reporting and found that IMUs wearable data was successful in their respective purposes and goals. As IMUs estimate translational and rotational body motions, we believe there is a strong potential for these applications to estimate velocity-dependent exaggeration of stretch reflexes and spasticity-related characteristics in spasticity. This review also proposes new directions of research that should be challenged by larger study groups and could be of interest to both researchers as well as clinicians. The use of IMUs to evaluate spasticity is a promising avenue to provide an objective measurement as compared to non-instrumented traditional assessments.

## 1. Introduction

Spasticity is a common syndrome in people with neurological impairments [1]. It is characterized by a velocity-dependent exaggeration of stretch reflexes and described by uncontrolled muscle overactivity that occurs when nerves operating muscle movement are demyelinated due to the disease process [2,3,4]. Spasticity may manifest in different motor dysfunctions as weakness, impairment of fine movements of the digits, hyperreflexia, loss of cutaneous reflexes, Babinski’s sign, clonus, spasms, and changes in posture [5], which all directly impact quality of life (QoL). The condition affects 85% of people with multiple sclerosis (MS), 35% with chronic hemiplegic stroke, and between 65% and 78% of people living with spinal cord injury (SCI) [6,7].

Measurement of spasticity is important to achieve effective management whether surgical, physical activity (e.g., stretching), pharmacologic (e.g., botulinum toxin focal injection), or instrumental (e.g., muscle vibration, bracing) [8,9,10]. Therefore, adequate assessment of spasticity is important in minimising the degree of disability [11].

One of the most common quantitative assessment techniques currently used to assess spasticity is based on a subjective ‘passive stretch’ performed by a clinician. The scale is based on a test that provides subjective results about muscle resistance to passive motions and requires no equipment from the evaluator.

Three examples of this technique include (1) the modified Ashworth scale (MAS), (2) the modified Tardieu scale (MTS), and (3) Fugl-Meyer assessment (FMA). These methods have been frequently used in clinics [12] because of their simplicity and ease of use [13,14]. Yet, the use of subjective scales has been strongly questioned due to their dependence on the expertise of the evaluator [15,16].

In addition, isokinetic dynamometry is a biomechanical method that has commonly been used for assessment and evaluation of spasticity since the 1980s [2,17,18]. It had been shown to produce reliable data when testing simple, uniaxial joints, such as the knee, with a great advantage of controlling the speed of exercise at a predetermined rate.

While several approaches are used in the evaluation and management of spasticity, physiotherapy techniques propose to recover motor performance partly through manipulation of muscle tone [19]. The Bobath approach [20], for example, advocates reduction of spasticity and developed postural reflexes through attention to trunk posture and controlled muscle stretch of the limbs [19].

The use of wearable motion sensors has proved to be an essential clinical tool within the healthcare sector and has become common practice in recent years [21]. Inertial measurement units (IMUs) allow estimation of kinematic parameters such as the body position, acceleration, and speed with high precision [22]. IMUs usually use biaxial or triaxial accelerometers to measure planar or 3D movement, respectively, gyroscopes to measure rotation, and magnetometers to assess relative position [23]. These devices incorporate aspects of traditional movement analysis techniques into everyday wearables and have the benefits of being low-power, pocket-sized, lightweight, and cost-effective, which make them attractive portable devices to different environments and assessment protocols. The role of such devices is twofold, from continuously recorded lab-based physical activity data for body motion analysis applications, to the Wearable Internet of Things (WIoT) which allows collection of a huge volume of personal health data, for use in health care systems at remote or local servers [24]. With the exponentially growing reputation of such devices [25], and their intrinsic ability to measure displacement and velocity of the body’s segments and joints, it is hard to overstate the magnitude of solutions this technology might help solve in spasticity management.

A new study [26] introduces a novel spasticity scale (SPAS), using two IMUs and EMG wearables in subjects with a complete spinal cord injury. The study was based on a complex model of the pendulum oscillation test [27] of the lower leg and was found to be highly correlated with the MAS technique. Likewise, Seoyoung et al. [28] proposed a novel IMU-based MTS assessment system to improve the accuracy and reliability of the method, with findings showing a significant improvement in the accuracy and reliability of MTS in lower limbs for children with cerebral palsy. In a third study, a novel system based on wearable EMG with IMU sensors incorporated visual feedback during MTS stretch reflex assessment [29]. The research concluded that the system could successfully capture clinically relevant features of elbow spasticity during stretch–reflex testing [30].

Although the use of these devices provides a promising objective and quantifiable outcome to the above traditional subjective techniques, prior review articles using wearable devices in different neurological disorders have reported poor standardised procedures and limited comparability across studies [30,31].

This systematic review aims to provide an updated framework of the current research in the use of IMUs-based wearable sensors in people living with spasticity.

Due to the rapidly volving nature of wearable technology, this review will focus on studies published in the past 5 years. In addition, the authors will provide their perspective on potential future applications in the field.

## 2. Methods

### 2.1. Search Strategy

A systematic literature search was performed to identify the most relevant quantitative and qualitative studies according to the Preferred Reporting Items for Systematic Reviews and Meta-Analyses (PRISMA) checklist [32]. The following electronic databases were searched: PubMed, Cochrane Library, and IEEE Xplore, to identify articles published from 1 January 2017 to 31 October 2021. The search terms combination used were (spasticity OR spastic* OR spasm) AND (wearable* OR assistive* OR apparel OR wearables OR sensors OR inertial* OR IMU OR assistive technology OR assistive device).

### 2.2. Study Selection Strategy

After detection and removal of duplicated manuscripts, two reviewers (Y.W. and O.T.) independently screened the title, abstract, and key words of the records identified through the database searching. If the record appeared relevant or if relevance was not immediately clear, the full text of the article was saved as a potential study to this review.

Literature administration was performed using RAYYAN [33], an online systematic review tool software.

Articles were included if they met the following criteria:

(1) Published in English.
(2) Full original research articles published in peer-reviewed scientific journals.
(3) Including adult human participants living with spasticity.
(4) Studies that focused on spasticity-related characteristics using IMU body-worn sensors in a clinical or community-based setting, or in “real life” environments.
(5) Wearable devices were portable, easy to use, and unobtrusive for the desired analysis.

We defined the following exclusion criteria in order to simplify the study selection and classification of the retrieved papers:

(1) Used animal models.
(2) Conference papers.
(3) No spasticity participant group was included.
(4) If study was not focused on IMU analysis as the prime tool.
(5) We also excluded studies focusing robot-assisted movement.

### 2.3. Data Extraction

Two reviewers (Y.W. and O.T.) studied the articles, and the following information was extracted into two tables: the study characteristics (Table 1) that include the aim, population type, selection criteria, and participants’ characteristics. The study parameters and outcome measures table (Table 2) includes IMU sensor type, body location, calculated parameters (i.e., a record of all variables computed from each wearable sensor signal), experimental protocol, study environment (e.g., indoor or outdoor), and key outcomes.

### 2.4. Methodological Quality

As this review represents a summary of recent studies that used wearable IMUs in people living with spasticity, conducted outside (day-to-day) and inside of the lab environments, the quality of each of the included articles was assessed using a custom quality assessment worksheet (Table 3). The reviews by Benson et al. and Campos et al. [8,34] were used as a basis for forming a quality assessment checklist. The quality assessment consists of 13 items distributed between four subscales including reporting, external validity, internal validity (bias), and power analysis. Two authors (Y.W. and O.T.) independently evaluated the methodological quality of each study included in this systematic review.

## 3. Results

### 3.1. Search Results

**Figure 1:**
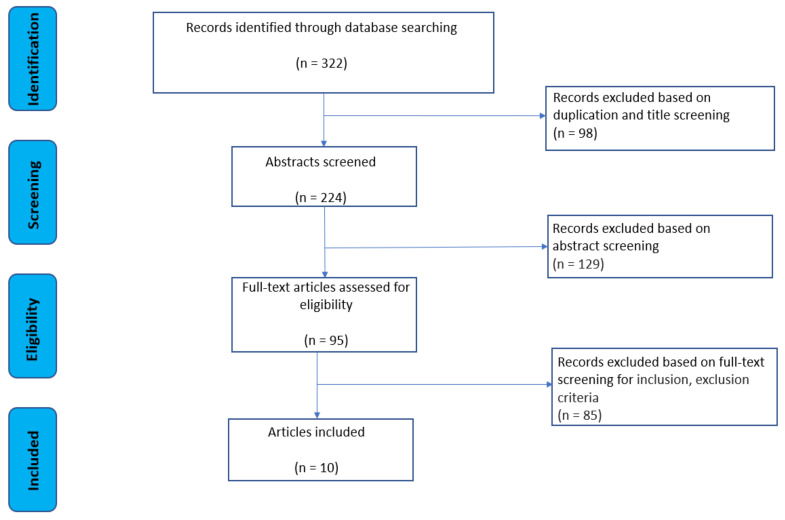
Strategy of literature review process.

The systematic review returned 10 [21,26,35,36,37,38,39,40,41,42] papers utilising between one to seven wearable IMUs for one or more explicit motion analysis purposes. The strategy of the literature review process and the selection of articles is presented in Figure 1. The initial literature search from the three databases yielded a total of 322 potential articles. After 98 duplicate references and title screening were removed, 224 citations remained for abstract screening. Next, 129 citations were rejected because they did not meet the inclusion criteria or met the exclusion criteria, and 95 citations were left for full text eligibility analysis. As a result, a total of 10 publications were included in this systematic review (Figure 1).

### 3.2. Study Characteristics

Table 1 shows the study and participant characteristics for all ten selected studies that used between one and seven IMU wearables for different purposes in people living with spasticity. Four studies [21,26,35,41] proposed a method or a framework for measurement of muscle spasticity level, and four other studies [37,39,40,42] focused on gait to calculate kinematic parameters in different environments (lab and real world). An additional study [38] investigated how severity of spasticity can affect quiet standing balance control, and another study [36] verified the relationship between commonly used clinical scales (FMA and MAS) and instrumented measurements. All studies were published in the past 5 years, between 2017 and 2021, and assessed different motion parameter measures. The total population sample size ranged from 6 to 86, with reported spasticity severity between 0–4 (MAS) and included mixed genders in their experimental phase. Out of the ten studies, six studies included chronic stroke patients [36,38,39,40,41,42], two studies included spinal cord injury patients [21,26], one study included multiple sclerosis patients [37], and another study included patients experiencing brain lesion [35].

Furthermore, while all selected studies reported either or both inclusion and excursion criteria, one study [41] did not explicitly account any of the study selection criteria based on these standards.

### 3.3. Study Parameters and Outcome Measures

Table 2 shows the summary of the parameters and outcome measures of the selected studies. To obtain data, all studies used at least one IMU sensor that was attached to the body and used different assessment protocols to collect data.

#### 3.3.1. IMU Details and Body Location

All studies clearly detailed their selected IMU brands. Seven studies [21,26,35,36,37,38,39] out of ten reported their sampling frequency, which ranged between 30–1000 Hz. Three studies [36,39,40] used BTS G-Walk sensor, one study [35] used MPU9250, InvenSense, one study [21] used Shimmer Sensing, one study [37] used Actigraph GT3X, one study [38] used Sway Star, Balance International Innovations GmbH, one study used [41] used APDM Opal, and one study [42] the RehaGait Pro Analyzer sensor. The locations of the wearable sensors on the body were reported by all studies to be placed at different points around the arm [21,35,37,41], the trunk [38,39,40,41,42], and at the lower limb [26,42]. All the images of the selected IMU brands are available online [43,44,45,46,47,48,49].

#### 3.3.2. IMU Assessment Protocol and Calculated Parameters

The data extracted from the IMUs were processed into variables that described the following characteristics during different assessment protocols. Two studies [21,35] conducted elbow movement tests to calculate the stretch angular velocity, angular acceleration, angle and peak angular acceleration, and other arm movement parameters (Table 2). One study [41] reported the upper limb joint torques using medium and fast arm speed tests of motion. Other studies [36,37,38,39,41,42] that conducted gait assessment protocols reported parameters such as the gait velocity, cadence, stride length, step length, step counts, vector magnitude counts, the level of physical activity intensity, three trunk angular velocities (yaw, roll, and pitch), gait cycle for a single stance phase, stand to sit and sit to stand accelerations, and other spatiotemporal parameters regarding the lower limb joint angles during walking and standing. Aleksić et al. [26] conducted an instrument pendulum test where the shank and foot were swinging freely to define the exponential fit to the spastic torque.

### 3.4. Methodological Quality

Table 3 summarizes the results of the methodological assessment for the included studies. All ten studies included within this review clearly described their respective state of the art, goals, participant characteristics, and findings. Selection criteria are fully described by six studies [36,38,39,40,41,42] that stated the inclusion and exclusion conditions. Yet, four studies [21,26,35,37] did not meet these standards as they did not explicitly indicate the exclusion or inclusion criteria in their methodology section. Regarding question number six, the estimations of random variability value were not adequately described by three studies [21,26,41]. Other studies [26,35,36] did not report actual probability values for the main outcomes, and all participants were representative of the populations being studied, i.e., subjects living with spasticity due to neurological disorder. In addition, all the studies had adequate test settings and conditions, and one study [37] was conducted in a real-life environment with the sensor located on the non-dominant wrist to measure continued physical activity data for seven days. The statistical tests and outcome measures were defined sufficiently in all studies except in Aleksić et al. [22], which is a clinical case study involving only six subjects, individually presenting their output measures. On the other hand, there was poor reporting on test–retest reliability and minimum detectable change values of the sensors, and only two studies [36,39] provided sample size justification, power description, or variance and effect estimates.

## 4. Discussion

Spasticity is a motor neuron syndrome described by velocity-dependent increase in muscle tone and uncontrolled reiterative involuntary contradictions of the skeletal muscle [50]. Although a broad range of traditional techniques are currently available for management spasticity, the use of wearable devices is increasingly recognized in the scientific community and holds a fertile ground for countless prospective applications in the field of spasticity. The purpose of this systematic review was to report the current state of use of IMUs-based wearable devices in people living with spasticity and to suggest future directions based on existing findings. This review illustrates how IMU wearable sensing technology has been used to investigate spasticity in different protocols and environments, including laboratory and free living. Due to several study objectives, methodologies, and key findings differences, a synthesis of evidence was impractical. The review also includes different population types as well as device specifications and assessment protocols used in recent studies. Table 4, adapted from Prill et al. [51], outlines the main study settings and characteristics for all selected manuscripts.

Our results strongly suggest that IMU wearable systems, often in combination with EMG sensor, show promise for quantifying spastic characteristics in clinical and day-to-day settings. We identified ten articles [21,26,35,36,37,38,39,40,41,42] published between 2017 and 2021 to be suitable in this review. Four studies [21,26,35,41] focused on proposing a novel method or framework to measure muscle spasticity. One of these studies [26] suggested a new scale—the spasticity scale (SPAS), based on the pendulum oscillation test [27], using two wearable IMUs and EMGs calculating the spasticity torque. The method is based on the level and the type of spasticity (flexion or extension) parameters and found to be highly correlated with the modified Ashworth score.

Study populations had different types of chronic illness disorders, with spasticity levels between 0–4 (MAS). This included chronic stroke, spinal cord injury, multiple sclerosis, and mixed brain lesion patients.

Seven off-the-shelf brands of IMUs were used to derive kinematic parameters, in which 30% used the BTS-G Sensor, and one a “home-made multi-channel signal recording system”. Surprisingly, the home-made device did not report enough technical information of their development, such as the type of microcontroller and related software, which could not be found anywhere else. We feel it is crucial that future studies involving non-commercial IMU-based wearables provide detailed technical information of their device, such as full size, weight, a basic diagram, type of microcontroller, and supporting software or integrated development environment (IDE). This approach could benefit future research and developers to replicate, validate, and improve the current state of the art for the favour of people living with spasticity.

All sensors were placed at different locations on the body at the upper limb, lower limb, and on the trunk, except for one study [36] that did not report any specific IMU placement position. Most commercial IMU wearable sensors weighed under 50 g, which makes them extremely light and attractive for long-term continuous-motion data-logging studies. On the other hand, Rahimzadeh et al. [38] used the SwayStar [49] which is a heavier device, weighing 750 g, to measure the trunk sway in balance control tests during quiet standing in people after stroke. This higher weight is not an issue as the SwayStar was developed to offer a tool for use in the examination of a patient’s stance and gait balance capabilities in more static positions [49] and has been shown to provide repeatable, accurate, and sensitive measures [52].

All studies reported applying between one to seven IMUs. Surprisingly, device-recording data frequency was only reported by seven papers [21,26,35,37,38,39]. The reported sampling rate ranged between 30–1000 Hz, which is appropriate as the protocols involved relatively slow movements. Nevertheless, we believe that not reporting apparatus manufacturer type or specific sampling rate frequency is a major quality assessment flaw and should be detailed and reported in future studies. Moreover, none of the studies calculated and reported the reliability of their apparatus, and only two studies [36,39] justified their selected sample size. All the above-mentioned studies showed success in demonstrating usability and feasibility of their respective proposed technique.

### 4.1. Future Directions

Only one of the ten studies reviewed was conducted out of a laboratory, in a day-to-day living environment. As we believe the great potential benefit of these devices is in home-based and day-to-day spasticity management, we propose several future directions for IMU use in this capacity:

- **Remote spasticity assessment**. A telehealth spasticity analysis platform for distant spasticity management, allowing for an alternative interaction between clinicians and patients. This system could combine EMG and IMU wearable sensors with feedback mechanisms to measure spasticity. The device, shaped into a sleeve, could be coupled with a guided motion assessment protocol. This approach could significantly improve the QoL of spasticity patients and reduce the burden of travel from their carers.
- **Focal muscle vibration (FMV) treatment system**. An auto-vibration treatment system based on EMG and IMU wearable sensors, as well as vibration motors, incorporated into a sleeve. The method could assess spasticity, in real time, and activate FMV to reduce symptoms and improve muscle functioning.
- **WIoT continuous data collection**. Using IMUs, uninterrupted kinematic spasticity muscle data mining could enable healthcare professionals and researchers to analyse the data for population health benefits.
- **A physiotherapy regime with real-time feedback**. Utilising a gaming framework, a stretching and sport regimen could be tailored to each patient. IMUs could provide real-time feedback to the user and clinician or record ongoing progress and outcomes.

### 4.2. Limitations

Several limitations should be acknowledged by the authors when interpreting the results of this review. We would firstly like to acknowledge the lack of homogenization of protocols in terms of clinical feasibility, study duration, and different environments (laboratory and day-to-day) which makes cross-study comparison very challenging. Next, the dates for inclusion were limited to the past 5 years, between 2017 to 2021. While this is an intentional selection criterion applied by the authors, allowing for the latest research to be highlighted, some impactful, cutting-edge studies published before 2017 may have been excluded.

## 5. Conclusions

During the last few years, many studies have been carried out to assess spasticity using wearable IMU sensors. This systematic review presents an updated overview on the use of IMUs-based wearable sensors in people living with spasticity in the past five years. Regardless of variations in study procedures, measured parameters, and some missing apparatus characteristics, the use of IMUs to evaluate velocity-dependent exaggeration of stretch reflexes and spasticity-related characteristics is a promising avenue to provide additional objective measurement as compared to non-instrumented traditional assessment techniques. The review also offers insights into potential future developments of the topic in an out-of-lab environment and may be of interest to both researchers and clinicians.

## Figures and Tables

**Table 1:** Study characteristics.

Author [Ref]	Aim	Population Type	Selection Criteria	Participants Characteristics
**Zhang et al., 2019**	To propose a novel regression-based framework for quantitative assessment of muscle spasticity using wearable surface EMG and IMU, combined with a simple examination procedure.	(1) Mixed: spinal cord injury, brain haemorrhage, brain trauma, brain infarction; (2) healthy subjects.	Inclusion: (a) participants experiencing stroke, acquired brain trauma, or incomplete spinal cord injury and accompanied by spasticity in flexor and extension muscles of the elbow; (b) the spasticity of elbow extensor or flexor was assessed within 1–3 grades using MAS; (c) the range of elbow joint during passive stretch was at least 120 degrees; (d) medically stable with clearance to participate; (e) without any historical musculoskeletal injuries or cognition problems; (f) able to offer informed signed consent prior to any procedure of the experiment. Exclusion criteria: n.a.	N: 16 (Spasticity); Gender: M/F: 14/2; Mean age: 54 ± 10; N: 8 (control); Gender: M/F: 6/2; Mean age: 29 ± 9; Spasticity severity (MAS): 1+–3.
**Jung-Yeon et al., 2020**	To propose a machine-learning based method, to provide information regarding the degree of spasticity of an elbow using a wearable device (IMU).	(1) CVA: cerebrovascular accident; (2) SCI: spinal cord injury.	Inclusion: Not explicitly mentioned. Exclusion: (1) Patients were excluded if their cognitive function was impaired (Mini-Mental State Examination score ≤ 23); (2) if they expressed discomfort in using a wearable device; (3) if the assigned therapist judged the participant to be unfit.	N:48; Gender: M/F: 26/22; Mean age: 61.2 ± 13.7 (M); 77.8 ± 10.1 (F); Spasticity severity (MAS): 0–4.
**Rech et al., 2020**	To verify the relationship between widely used clinical scales FMA and MAS and instrumented measurements to evaluate poststroke individuals with motor impairment.	Chronic hemiparesis after stroke.	Inclusion: (1) Adults aged between 19 and 80 years; (2) history of unilateral cortical or subcortical stroke diagnosis confirmed by brain imaging exam (tomography or magnetic resonance); (3) time since stroke from 6 months to 5 years; 4) ability to reach 60 degrees of shoulder flexion with the paretic upper limb and walk for at least 10 m (with or without walking devices). Exclusion: (1) Individuals were excluded if they presented: cerebellum lesion; (2) musculoskeletal disorders that could impair the reaching task and/or gait performance; (3) cognitive impairment (<20/30 points illiterate or <24/30 points in the Mini-Mental State Examination 15).	N: 34; Gender: M/F: 23/11; Mean age: 58.38 ± 14.56; Spasticity severity (MAS): 0–4.
**Pau et al., 2020**	(1) To investigate the existence of possible differences between women and men with MS in terms of amount and intensity of PA performed during a week, continuously acquired using wrist-worn wearable accelerometers and (2) to verify whether the disease has a stronger impact on men or women with MS.	(1) Multiple sclerosis group; (2) control group.	Inclusion (MS): (1) diagnosis of MS, (2); age between 18 and 65 years; (3) Expanded Disability Status Scale (EDSS, a score used to quantify the disability caused by MS based on a neurological examination of 8 functional systems) score ≤ 6; (4) being clinically stable and on treatment with disease-modifying agents for at least 6 months. Inclusion (control): 41 age-matched unaffected individuals (21 women, 20 men), selected among relatives and caregivers of the pwMS and hospital staff, composed the control group. Exclusion: n.a.	N: 45 (MS); Gender: M/F: 22/23; Mean age (MS): 51.2 ± 11.8 (M); 49.4 ± 9.0 (F); N: 41 (Control): Gender: M/F: 20/21; Mean age: 49.6 ± 14.4 (M); 46.7 ± 14.6 (F); Spasticity severity (MAS): n.a.
**Rahimzadeh et al., 2017**	To investigate how severity of spasticity can affect quiet standing balance control in individuals following stroke.	Participants with stroke: (1) individuals with low plasticity (MAS score < 2); (2) individuals with high plasticity (MAS score > 2).	Inclusion: (1) the ability to stand independently, with and without; eyes open for at least 10 min. Exclusion: (1) inability to stand unassisted, (2) inability to follow simple instructions due to cognitive impairments as determined; by clinicians, (3) fixed ankle contracture, 4) or being treated with botulinum toxin injections within the past 3 months of study participation.	N: 12 (low spasticity); Gender: M/F: 8/4; Mean age: 74.3 ± 3.4; Spasticity severity (MAS): 0–3; N: 15 (high spasticity); Gender: M/F: 11/4; Mean age: 61.8 ± 3.0.
**Aleksić, Antonina, and Dejan B. popović, 2021**	To develop a simple quantitative objective measure of spasticity, based on pendulum test	Complete spinal cord injury.	Inclusion: (1) complete lesion above the Th12; (2) stable neurological and medical status; no autonomic dysreflexia; (3) no cognitive disorders; and (4) no medical history of hearing or balance disorders. Exclusion: n.a.	N: 6; Gender: M/F: 3/3; Age: 25–58 years old; Spasticity severity (MAS): 0–4.
**Garcia et al., 2021**	To explore a novel movement quality metric, the estimation of gait smoothness by the spectral arc length (SPARC), in individuals with a chronic stroke displaying mild/moderate or severe motor impairment while walking in an outdoor environment.	(1) Chronic stroke group; (2) Control group	Inclusion (stroke): (1) aged between 18 and 80 years; (2) with a diagnosis of cortical or subcortical unilateral cerebrovascular accident confirmed by imaging; (3) time since the stroke from 6 months to 10 years; (4) ability to walk at least 10 m with or without assistive devices, and (5) minimum score of 20/30 points (illiterate) or >24/30 points (literate) in the Mini-Mental State Examination (MMSE). Exclusion (stroke): (1) clinical diagnosis of musculoskeletal diseases, (2) significant visual deficit, and (3) history of falls in the last 3 months. Exclusion (control group): previous history of neurological or musculoskeletal disorders that induced visible gait abnormalities.	N: 32 (control); Gender: M/F: 22/10; Mean age: 56.81 ± 8.88; N: 32 (stroke); Gender: M/F: 22/10; Mean age: 56.84 ± 9.10; Spasticity severity (MAS): 0–4.
**Kowal et al., 2020**	To evaluate the temporospatial parameters of gait and assessed the maximal isometric and isokinetic torque production of the plantar flexor and dorsiflexor muscles.	(1) Stroke; (2) control group.	Inclusion (spasticity group): (1) the normal range of motion values for both ankle joints, (2) no decreased strength of the muscles acting on ankle joints in a physical examination, and (3) no cognitive or mental disorders. Exclusion (spasticity group): (1) a level of spasticity higher than grade one on the MAS, (2) severe limb pain, (3) sensory impairment, (4) visual impairment, (5) cognitive impairment, (6) balance disturbances, (7) the presence of other neuromuscular or musculoskeletal disorders, and (8) the inability to independently walk a distance of at least ten metres.	N:15 (control); Gender: M/F: 7/8; Mean age: 32.3 ± 4; N:15 (stroke); Gender: M/F: 7/8; Mean age: 57.2 ± 11; Spasticity severity (MAS): 0–1.
**Ang et al., 2018**	To present a method based on a human upper limb model that assesses the severity of spasticity in patients with stroke objectively	Stroke.	Inclusion: n.a.; Exclusion: n.a.	N: 15; Gender: M/F: 7/8; Mean Age: 56.9 ± 10.4; Age: 32–75 years old; Spasticity severity (MAS): 0–1+.
**Varvarousis et al., 2021**	to provide evidence of the beneficial effect of intramuscular BoNT-A injections on characteristics of gait pattern on patients suffering from upper motor neuron lesion with equinovarus deformity, particularly with regards to spatiotemporal parameters.	Post-stroke.	Inclusion: (1) age from 18 to 75 years, (2) patients had to be able to walk either freely or while wearing a splint or by the use of a crutch, (3) level of spasticity ≥1 + on the modified Ashworth scale, (4) poststroke period at least 6 months and nonsurgical operation on the lower extremities. Exclusion: (1) patients with dementia or aphasias, (2) history of previous injection of BoNT-A the last six months, (3) fixed joint posture (contraction), and (4) hospitalization and pregnancy.	N: 13; Gender: M/F: 9/4; Age: 37–73 years old; Spasticity severity (MAS): 1+–3.

**Table 2:** Study parameters and outcome measures.

Author [Ref]	IMU Sensor Type & Specifications	Location/s	Calculated Parameters From IMU	IMU Assessment Protocol	Environment	Main Findings
**Zhang et al., 2019**	MPU9250, InvenSense (“home-made system”); 100 Hz; dimensions: n.a.; weight: n.a.	Ipsilateral medial wrist.	Elbow stretch angular velocity, angle, angular acceleration, peak angular acceleration.	2 × (15 to 20 trials of 3–7 s each) of passive elbow stretch with different velocities. Subjects were instructed to fully extend the tested elbow at 180 degrees with palm upward. Then, the tested elbow was passively pulled by an experimenter to elbow flexion at 40–60 degrees with a stretch range of 120–140 degrees. After a 2-s pause, it was passively stretched back to 180 degrees. The stretch velocity was determined subjectively in each trial by the experimenter and kept to almost constant during the stretch.	Controlled.	The experimental results demonstrated the usability and feasibility of the proposed framework, and it provides an objective, quantitative and convenient solution to spasticity assessment, suitable for clinical, community, and home-based rehabilitation. The suggested model showed a moderate goodness of fit (R2 = 0.4990, *p* < 0.001) to the MAS grades.
**Jung-Yeon et al., 2020**	Shimmer Sensing; 256 Hz; 51 × 34 × 14 mm; 23.4 g.	Dorsal side of the affected elbow or, the dominant side of the elbow for no spastic symptom subjects.	Acceleration and angular velocity parameters (during elbow stretch): (1) root mean square, (2) mean, (3) standard deviation, (4) energy, (5) spectral energy, (6) absolute difference, (7) variance extracted from pitch and roll, (8) signal magnitude area (SMA) and, (9) signal vector magnitude (SV).	The therapist held the affected arm of a participant still (quasi-static state) to stabilize signals of IMUs, then had the elbow moved by one cycle per second.	Controlled.	A machine-learning algorithm, random forest (RF), performed well, achieving up to 95.4% accuracy. Findings demonstrated how wearable technology and machine learning can be used to generate a clinically meaningful index but also offers rehabilitation patients an opportunity to monitor the degree of spasticity, even in nonhealthcare institutions where the help of clinical professionals is unavailable.
**Rech et al., 2020**	BTS G-WalkSampling rate:100 Hz; 70 × 40 × 18 mm; 37 g.	S1 vertebrae	LL (motor) test IMU: gait velocity (m/s), cadence (steps/min), stride length (m), and step length (m).	LL test: 10 m walking test and the Timed up and go test (TUG) and accelerations during the sit to stand check.	Controlled.	FMA correlated with motor performance (upper and lower limbs) and with movement quality (upper limb). Modified Ashworth scale correlated with movement quality (upper limb). Cut-off values of 9.0 cm in trunk anterior displacement and 57 m/s in gait velocity were estimated to differentiate participants with mild/moderate and severe compromise according to the FMA. Gait parameters measured by the IMU showed a moderate correlation with severity of motor function and level of spasticity. These results suggest that the FMA can be used to infer about motor performance and movement quality in chronic poststroke individuals with different levels of impairment.
**Pau et al., 2020**	Actigraph GT3X; sampling rate at 30 Hz; 38 × 37 × 18 mm; 27 g.	Non-dominant wrist (acceleration).	(1) Step counts (SC); (2) vector magnitude counts (VM); (3) levels of PA intensity (classified into three categories).	Day to day physical activity (PA), for 7 consecutive days.	Day-to-day.	Women with MS spent more time in sedentary behaviour and exhibited a reduced amount of light intensity activity with respect to men, while MVPA was similar across sexes. However, in comparison with unaffected individuals, the overall PA patterns appear significantly modified mostly in women who, in presence of the disease, present increased sedentary behaviour, reduced MVPA, number of daily steps and VM counts. The number of daily steps calculated from IMU for both women and men regardless of disability level was 9032 steps/day.The findings of the present study highlight the urgency of including sex as variable in all studies on PA in pwMS.
**Rahimzadeh et al., 2017**	SwayStar, Balance International Innovations GmbH; 100 Hz; 150 × 110 × 90 mm; 750 g.	Mounted near the lumbar region of the trunk.	(1) Trunk sway (angular velocity) (2) displacement in the pitch (anterior–posterior) and (3) roll (mediolateral) directions.	Altering order of: (1) 2 × (eyes open: participants stood still as possible for 80 s on force plate) and (2) 2 × (eyes close: participants stood still as possible for 80 s on force plate).	Controlled.	The high spasticity group demonstrated greater ML COP velocity, trunk roll velocity, trunk roll velocity frequency amplitude at 3.7 Hz, and trunk roll velocity frequency amplitude at 4.9 Hz (measured by the IMU), particularly in the eyes closed condition (spasticity by vision interaction). ML COP MPF was greater in the high spasticity group. Individuals with high spasticity after stroke demonstrated greater impairment of balance control in the frontal plane, which was exacerbated when vision was removed.
**Aleksić, Antonina, and Dejan B. popović, 2021**	2 × IMU: 3F-FIT FABRICANDO FABER; sampling at 1 kHz; dimensions: n.a.;weight: n.a.	(1) Thigh; (2) shank.	(1) A defining the exponential fit to the spastic torque (angular velocity and angle); (2) the new measure spasticity scale SPAS.	(1) Spasticity assessment: modified Ashworth scale. (2) pendulum test: the examiner released the lower leg from the knee joint’s full extension, and the shank and foot were swinging freely.	Controlled.	Introduction of new spasticity scale (SPAS), which was found highly correlated with the modified Ashworth score.
**Garcia et al., 2021**	BTS G-Walk; sampling at 100 Hz; 70 × 40 × 18 mm; 37 g.	Attached to the subjects’ waists covering the L5 S1 segments.	The trunk angular velocities (yaw, roll, and pitch) (in °/s).	(1) Lower limb motor impairment: Fugl-Meyer Assessment (2) MAS was used to evaluate resistance to passive movements. (3) Gait assessment: walk at self-selected speed on a 10 m pathway.	Controlled.	Individuals with a chronic stroke displayed reduced smoothness in the yaw and roll angular velocities while walking in an outdoor environment. The IMU parameters showed that mild and moderate stroke group presented less smooth-ness gat than the control group (*p* = 0.015). The quantification of gait smoothness using the SPARC metric may represent an additional outcome in clinical assessments of gait in individuals with a chronic stroke.
**Kowal et al., 2020**	BTS G-Walk; Sampling rate: n.a.; 70 × 40 × 18 mm; 37 g.	(1) at the level of the sacral bone (S1) for gait analysis (2) TUG: the level of the lumbar spine (L2).	(1) (a) Gait cadence (GCAD) [steps/min], (b) gait speed (GSP) [m/s], (c) gait cycle (GC) for the single stance phase [%] (d) (2) (a) stand-to-sit VTA [m/s²], (b) sit-to-stand VTA [m/s²] between the post-stroke and control groups.	Gait analysis, 7 m walk with self-selected speed. Repetitions of this movement task were recorded for further analysis of the mean course and to extract the pattern of representative data, following by TUG test.	Controlled.	Post-stroke patients had statistically significantly lower gait cadence than healthy participants (17%, *p* < 0.05). Statistically significantly lower values of vertical acceleration were also noted during a sit-to-stand movement task (42%, *p* < 0.05). In nutshell, Despite the low intensity of spasticity and early phase after stroke, differences in the muscle torque production and temporo-spatial parameters, as well as the correlations between them, were noticeable.
**Ang et al., 2018**	3 × IMUs (APDM Opal™ wireless); sampling rate: n.a.; 43.7 × 39.7 × 13.7 mm; 25 g.	Upper limb: (1) flat portion of the sternum, just below the neck (2) middle part of the upper arm and, (3) lower arm.	Upper limb joint torques calculated by measured joint angles velocities and accelerations using IMUs.	Medium and fast arm speed tests of motion. The patient sat on a chair with the arms resting in a relaxed position beside the body for 5 s before the therapist held up the patient’s arm. The therapist then extended the patient’s elbow in 2 to 3 s for slow speeds, in 1 to 2 s for medium speeds, and as quickly as possible without causing pain to the patient for fast speeds. For each speed range, the tests were performed four times with one-minute rest after two repetitions. A total of 12 tests were carried out for each patient.	Controlled.	The estimated muscle activation profiles, calculated by measured joint angles velocities and accelerations data obtained by IMUs, have a high correlation (0.707) to the EMG signal profiles. The null hypothesis that the rankings of the severity using the model and the MAS assessment have no correlation has been tested and was rejected convincingly (*p* ≈ 0.0003). These findings suggest that the model has the potential to complement the existing practices by providing an alternative evaluation method.
**Varvarousis et al., 2021**	7 × RehaGait Pro Analyzer; sampling rate: n.a.; 60 × 15 × 35 mm; 34 g.	(1) Ankle joints, (2) calves, (3) thighs and (4) at the theoretical body centre of mass.	Spatiotemporal specific parameters during walking and standing: (1) Min Ankle, (2) Max Ankle angle, (3) Min knee angle, (4) Max knee angle, (5) Heel Strike angle, (6) Toe Off angle, (7) Max foot height, and (8) Max circumduction.	(a) Gait: (IMU) the patient had to walk, in a self-chosen speed, across a walkway covering at least 15 m. The procedure was repeated 4 times, with short intervals between the repetitions if the patient felt fatigue or dizziness.	Controlled.	Comparison of the parameters calculated from the IMU, between normal and hemiplegic lower extremity before BoNT-A injection showed statistically significant differences for the parameters: Max Ankle angle (*p* = 0.033), Max Knee angle (*p* = 0.006), Max foot height (*p* = 0.008) and Max circumduction (*p* = 0.013), Min Ankle angle (*p* = 0.006), Max Ankle angle (*p* = 0.039), Max Knee angle (*p* = 0.007), Max foot height (*p* = 0.004) and Max circumduction (*p* = 0.033). While all spatio-temporal parameters of motion analysis and balance improved for most of the patients after botulinum toxin injection, only one parameter, the normal to hemiplegic step length, reached statistically significant improvement (*p* < 0.03). Moreover, the modified Ashworth score was statistically improved post injection (*p* < 0.001). In conclusion the use of botulinum toxin injections is beneficial in post stroke patients as this is depicted in gait parameters improvement which accompanies the spasticity reduction.

**Table 3:** Quality assessment questions.

Question	Zhang et al., 2019	Jung-Yeon et al., 2020	Rech et al., 2020	Pau et al., 2020	Rahimzadeh et al., 2017	Aleksić, Antonina, and Dejan B. Popović., 2021	Garcia et al., 2021	Kowal et al., 2020	Ang et al., 2018	Varvarousis et al., 2021
**Q1.** Is the hypothesis/aim/objective of the study clearly described?	Y	Y	Y	Y	Y	Y	Y	Y	Y	Y
**Q2.** Are the clearly described in the Introduction or Methods?	Y	Y	Y	Y	Y	Y	Y	Y	Y	Y
**Q3.** Are the characteristics of the participants clearly described (including age, sex, and status as healthy/injured/pathological)?	Y	Y	Y	Y	Y	Y	Y	Y	Y	Y
**Q4.** Are the inclusion/exclusion criteria described and appropriate?	N	N	Y	N	Y	N	Y	Y	Y	Y
**Q5.** Are the main findings of the study clearly described?	Y	Y	Y	Y	Y	Y	Y	Y	Y	Y
**Q6.** Are estimates of the random variability in the data for the main outcomes provided?	Y	N	Y	Y	Y	N	Y	Y	N	Y
**Q7.** Have actual probability values been reported for the main outcomes?	N	Y	N	Y	Y	N	Y	Y	Y	Y
**Q8.** Are the participants representative of the entire population from whichthey were recruited?	Y	Y	Y	Y	Y	Y	Y	Y	Y	Y
**Q9.** Are the setting and conditions typical for the population represented by the participants?	Y	Y	Y	Y	Y	Y	Y	Y	Y	Y
**Q10.** Are the statistical tests used to assess the main outcomes appropriate?	Y	Y	Y	Y	Y	N	Y	Y	Y	Y
**Q11.** Are the main outcome measures used accurate (valid and reliable)?	Y	Y	Y	Y	Y	Y	Y	Y	Y	Y
**Q12.** Have test-retest reliability and minimum detectable change values of the sensors reported?	N	N	N	N	N	N	N	N	N	N
**Q13.** Is a sample size justification, power description, or variance and effect estimates provided?	N	N	Y	N	N	N	Y	N	N	N

Note: Y = yes, N = no.

**Table 4:** Summary of IMU characteristics and methods.

	Zhang et al., 2019	Jung-Yeon et al., 2020	Rech et al., 2020	Pau et al., 2020	Rahimzadeh et al., 2017	Aleksić, etal., 2021	Garcia et al., 2021	Kowal et al., 2020	Ang et al., 2018	Varvarousis et al., 2021
**Number of IMU Sensors**	1	1	1	1	1	2	1	1	3	7
**Commercial Device**		+	+	+	+	+	+	+	+	+
**Sampling Frequency: <50 Hz**				+				N.R.	N.R.	N.R.
**Sampling Frequency: 50–200 Hz**	+			+	+		+	N.R.	N.R.	N.R.
**Sampling Frequency: 200–1000 Hz**		+	+			+		N.R.	N.R.	N.R.
**Weight: <50 g**	N.R.	+	+	+		N.R.	+	+	+	+
**Weight: >500 g**	N.R.				+	N.R.				
**Upper Limb Placement**	+	+		+	+					
**Lower Limb Placement**						+			+	+
**Trunk Placement**			+				+	+		+
**Controlled Environment**	+	+	+		+	+	+	+	+	+
**Day–Day environment**				+						

+ = Yes; N.R. = not reported.

## Data Availability

No data were generated.
